# Different Patterns of Punctate White Matter Lesions in Serially Scanned Preterm Infants

**DOI:** 10.1371/journal.pone.0108904

**Published:** 2014-10-03

**Authors:** Karina J. Kersbergen, Manon J. N. L. Benders, Floris Groenendaal, Corine Koopman-Esseboom, Rutger A. J. Nievelstein, Ingrid C. van Haastert, Linda S. de Vries

**Affiliations:** 1 Department of Perinatology, Wilhelmina Children's Hospital and Brain Center Rudolf Magnus, University Medical Center Utrecht, Utrecht, The Netherlands; 2 Department of Radiology, University Medical Center Utrecht, Utrecht, The Netherlands; University of Pécs Medical School, Hungary

## Abstract

**Background and Purpose:**

With the increased use of MRI in preterm infants, punctate white matter lesions (PWML) are more often recognized. The aim of this study was to describe the incidence and characteristics of these lesions as well as short-term outcome in a cohort of serially scanned preterm infants, using both conventional imaging, diffusion (DWI) and susceptibility (SWI) weighted imaging.

**Materials and Methods:**

112 preterm infants with 2 MRIs in the neonatal period, with evidence of punctate white matter lesions, were included. Appearance, lesion load, location, and abnormalities on DWI and SWI were scored and outcome data were collected.

**Results:**

Different patterns of punctate white matter lesions did appear: a linear appearance associated with signal loss on SWI, and a cluster appearance associated with restricted diffusion on DWI on the first MRI. Cluster and mixed lesions on the first scan changed in appearance in over 50% on the second scan, whereas linear lesions generally kept their appearance. Lesions were only visible on the early scan in 33%, and were only seen at term equivalent age in 20%. Nine infants developed cerebral palsy, due to additional overt white matter lesions in six.

**Conclusion:**

Two patterns of punctate white matter lesions were identified: one with loss of signal on SWI in a linear appearance, and the other with DWI lesions with restricted diffusion in a cluster appearance. These different patterns are suggestive of a difference in underlying pathophysiology. To reliably classify PWML in the preterm infant in either pattern, an early MRI with DWI and SWI sequences is required.

## Introduction

With the increased use of MRI in preterm infants, more subtle punctate lesions and diffuse white matter signal abnormalities are increasingly recognized [1], [2]. The incidence of these punctate white matter lesions (PWML) varies widely, from less than 10 to over 50% [1], [3]–[7]. Identification of risk factors responsible for PWML has proven to be difficult and data on outcome are controversial [8]–[10]. Most studies so far have either used conventional T1-weighted and T2-weighted imaging or diffusion tensor imaging to study PWML. Only a few studies have utilized additional sequences more sensitive to blood, blood products, or gliosis [11]–[13].

Obtaining a better understanding of the extent, appearance and possible underlying pathophysiology of PWML is important for accurate counseling of parents of preterm infants, and for a better selection of those infants that may benefit from neuroprotective strategies or early rehabilitation services.

The aim of this study was to describe the appearance, location and evolution of PWML in a cohort of serially scanned preterm infants utilizing conventional imaging as well as diffusion weighted imaging (DWI) and susceptibility weighted imaging (SWI), and to correlate these findings with neurodevelopmental outcome.

## Methods

For this study, all infants born below a gestational age (GA) of 34 weeks and admitted to the Neonatal Intensive Care Unit of the Wilhelmina Children's Hospital between March 2007 and March 2013, with at least two MRI scans in the neonatal period, were retrospectively identified. The group of included infants consisted of two subgroups. Infants with a GA<28 weeks were routinely scanned based on their extreme prematurity, whereas infants with a GA≥28 weeks were only scanned when serial cranial ultrasound examinations were suggestive of brain injury. Infants were included in the present study if they had been reported to have PWML on one or both scans. A total of 119 infants were identified. Seven infants had congenital anomalies and were therefore excluded. The remaining 112 infants were eligible for this study. Permission from the medical ethical review committee of the University Medical Center Utrecht (MERC UMC Utrecht) for the current study and informed parental consent for the MRI was obtained. Patient data were anonymized prior to analysis. Since this was a retrospective study, using MRIs performed as part of standard clinical care, oral consent for the MRI was obtained by the treating physician and any questions or remarks were noted in the charts. No written consent was deemed necessary. The MERC UMC Utrecht waived the need for parental consent for both the use of medical data and for publication of medical images.

### Magnetic Resonance Imaging

In the subgroup with a GA<28 weeks, MRI was performed around 30 weeks postmenstrual age and again around term equivalent age (TEA). The subgroup with a GA≥28 weeks was scanned as soon as possible after birth and again around TEA.

MRI was performed on either a 1.5 Tesla ACS-NT system (early n = 9, TEA n = 3) or a 3 Tesla (early n = 103, TEA n = 109) whole-body Achieva system (Philips Medical Systems, Best, the Netherlands). On the 1.5T magnet, the routine protocol included conventional inversion recovery-weighted imaging and T2-weighted imaging (30 and 40 weeks: inversion recovery-weighted repetition time [TR] 4147 ms; inversion time [TI] 600 ms; echo time [TE] 30 ms; slice thickness 2 mm and T2-weighted TR 7656 ms; TE 150 ms; slice thickness 2 mm), as well as DWI (single-shot echo planar imaging in 3 orthogonal directions; 25 slices; slice thickness 4 mm; TR 3700–5200 ms; TE 89 ms; b-values of 0 and 1000 mm^2^/s, no gap).

On the 3T magnet, the routine protocol included conventional 3D T1-weighted and T2-weighted imaging (30 weeks: 3D T1-weighted TR 9.4 ms; TE 4.6 ms; slice thickness 2 mm and T2-weighted TR 10085 ms; TE 120 ms; slice thickness 2 mm; 40 weeks: 3D T1-weigthed TR 9.5 ms; TE 4.6 ms; slice thickness 1.2–2 mm and T2-weighted TR 4847–6293 ms; TE 120–150 ms; slice thickness 1.2–2 mm), as well as DWI (single-shot echo planar imaging in 3 orthogonal directions; 25 slices; slice thickness 4 mm; TR 2393 ms; TE 68 ms; b-values of 0 and 800 mm^2^/s, no gap).

From 2008 onwards, the SWI sequence became available (3D gradient-echo sequence with flow compensation, multishot echo-planar imaging; at 1.5T: TR 82 ms; TE 40 ms, at 3T: TR 52 ms; TE 30 ms, slice thickness 2 mm and EPI factor 3 in both). Since this sequence was not included in the standard protocol, use was at the discretion of the attending neonatologist and dependent on the time left in the scanning timeslot.

Infants were sedated using oral chloral hydrate 30 to 60 mg/kg. Heart rate, transcutaneous oxygen saturation (Nonin Medical Incorporated, Minneapolis, MN, USA) and respiratory rate were monitored. For hearing protection Minimuffs (Natus Medical Incorporated, San Carlos, CA, USA) and Earmuffs (EM's 4 Kids, Brisbane, Australia) were used. A neonatologist or physician assistant was present throughout the examination.

### Clinical and follow-up data

Clinical data were retrieved from the medical records. All infants were seen according to clinical protocol in our follow-up clinic. At 15 months corrected age, the Griffiths Mental Development Scales were used to assess neurodevelopmental outcome [14]. Around 24 months corrected age, either the Griffiths Mental Development Scales or, in infants with a GA<28 weeks, the Bayley Scales of Infant and Toddler Development, third edition were used [15]. The presence of cerebral palsy (CP), epilepsy, visual or hearing problems was also recorded.

### Re-evaluation of the data

All scans were re-evaluated (KJK) using a combination of T1-weighted imaging, T2-weigthed imaging, DWI and –when available– SWI. PWML were defined as small areas of high signal intensity (SI) on T1-weighted imaging and low SI on T2-weighted imaging. The number of PWML as well as the appearance, location and laterality were scored, with a system based on that of Cornette [5]. For early MR scans, the number of lesions was divided in <3, <6 and ≥6 or containing over 5% of a hemisphere, in agreement with the literature [3]. For TEA scans, a division of more or less than 6 lesions was used.

**Figure 1:**
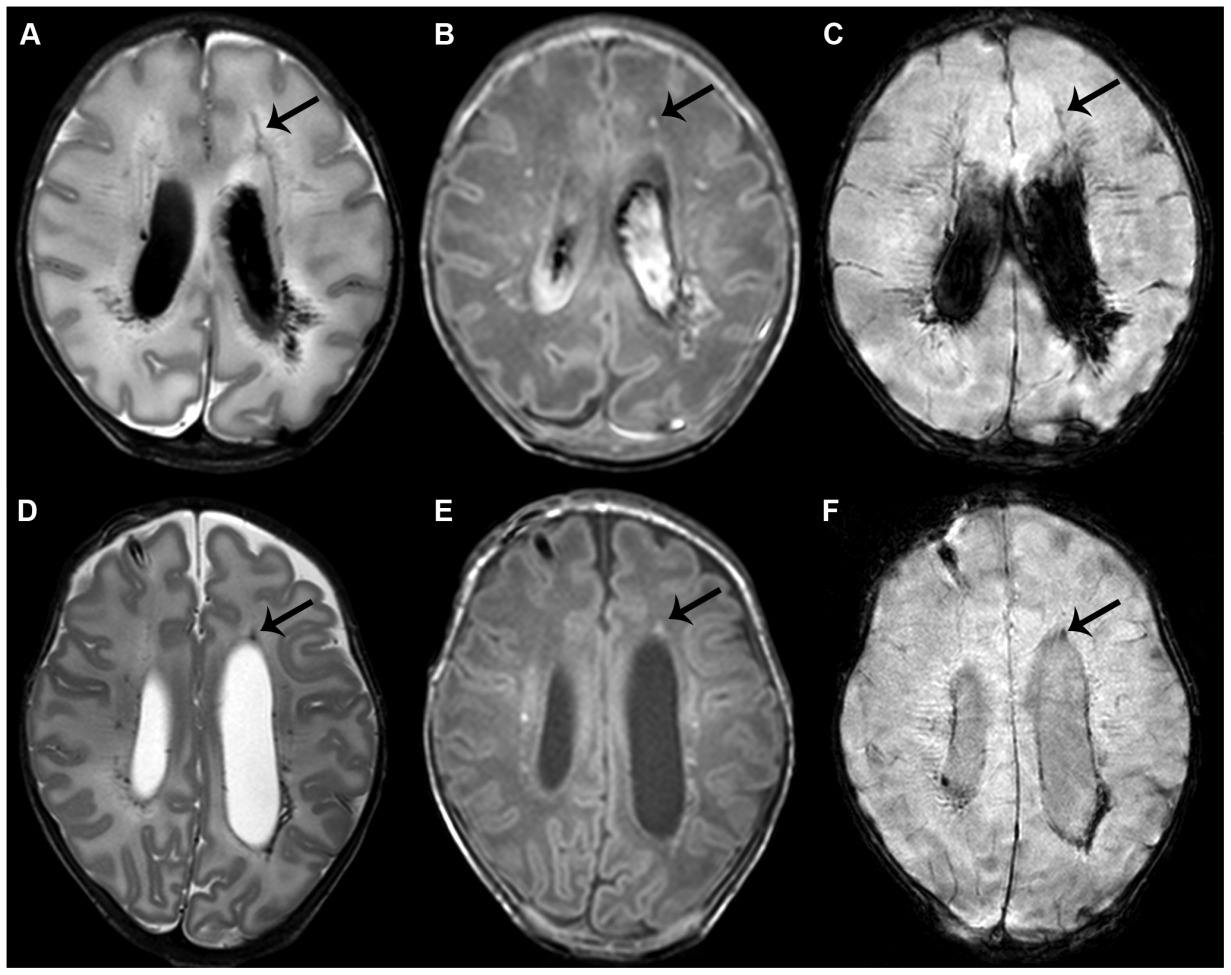
Punctate white matter lesions with a linear pattern. Early (A–C) and term equivalent (D–F) scans in an infant of 32 weeks gestation with lesions in a mixed, though mainly linear pattern. T2-weighted imaging shows bilateral lesions in the white matter adjacent to the ventricles (A). Lesions are also visible on T1-weighted imaging, additionally showing a small subdural hemorrhage (B). The arrow indicates one of the lesions, visible on all images. Also note the grade III intraventricular hemorrhage. SWI shows signal loss in the areas of the lesions, suggestive of a hemorrhagic origin (C). Additionally, some small frontal lesions can be identified, that do not show up on SWI, and show a cluster appearance. At term equivalent age, lesion load has greatly diminished and the intraventricular hemorrhage has largely resolved on T2- and T1-weighted imaging (D, E). A subcutaneous reservoir has been inserted to treat post-hemorrhagic ventricular dilatation. SWI still shows signal loss with blood residue being most clearly visible on this sequence (F). Outcome was favorable with a cognitive composite score of 115 and a total motor composite score of 124 on the Bayley scales at two years corrected age.

**Figure 2:**
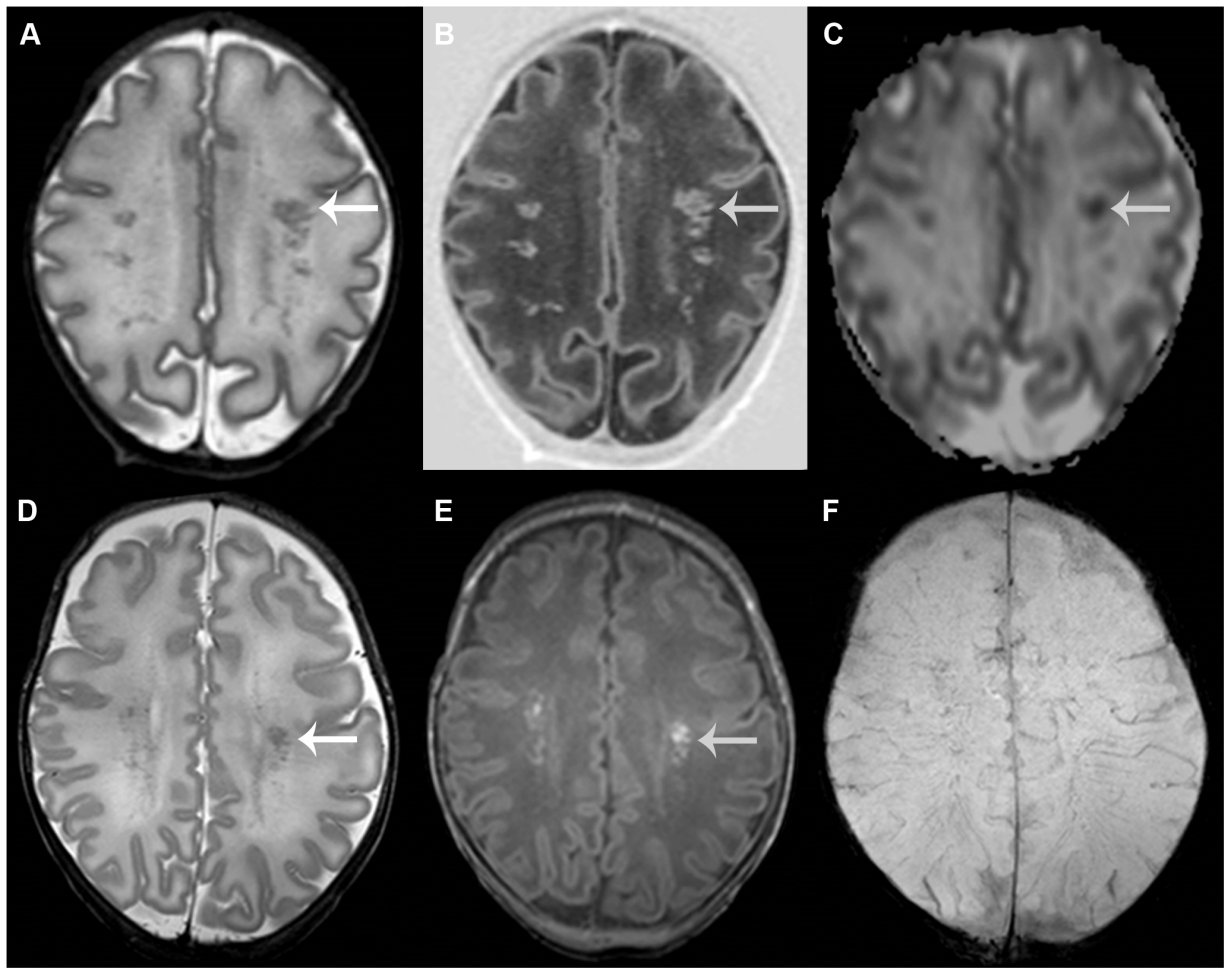
Punctate white matter lesions with a cluster pattern. Early (A–C) and term equivalent (D–F) scans in an infant of 31+3 weeks gestation with lesions in a cluster pattern. T2-weighted imaging shows multiple lesions throughout the white matter (A) that are also clearly visible on inversion recovery imaging (B). The apparent diffusion coefficient -map shows restricted diffusion at the site of the lesions (C). The SWI sequence (not shown) did not show any signal loss. The arrow indicates one of the lesions, visible on all images. At term equivalent age, lesion load has diminished. Lesions are now better appreciated on T1-weighted imaging (E), compared with T2-weighted imaging (D). Again, SWI does not show signal loss (F). Outcome was favorable with a developmental quotient of 94 on the Griffiths scales at 18 months corrected age.

Appearance was divided in lesions with a linear appearance, an appearance of (solitary) clusters or a mixed appearance containing a combination of both. *Linear appearance* was defined as multiple lesions in close relation to another, with a linear organization, often in close approximation to the ventricles. A *cluster appearance* was defined as more solitary lesions, with a rounded shape and somewhat larger in size, which were often, although not exclusively, located deeper in the white matter (see *Figure 1* and *Figure 2* for examples). Location was divided in anterior, mid and posterior to the lateral ventricles and laterality was scored as unilateral or bilateral involvement. The DWI sequences were evaluated on the presence of lesions with restricted diffusion. When available, the SWI sequences were evaluated on the presence of foci of signal loss outside the ventricles or the ventricular wall, which did not have continuity suggestive of veins or venous congestion.

Scans were also scored for the presence of intraventricular hemorrhage (IVH) and other overt white matter lesions. In infants with additional lesions, such as a unilateral periventricular hemorrhagic infarction (PVHI), only PWML outside the area of the parenchymal lesion were scored.

### Statistical analyses

Statistical procedures were performed using R version 2.15.3 (www.r-project.org). First, chi square tests were used to assess the relation between findings of PWML on the DWI or SWI sequence and the appearance of PWML, the relation between IVH and appearance, and to assess the effects of GA, and thereby the possible inclusion bias due to the two subcohorts. The subcohorts, defined as having a GA above or below 28 weeks, were tested against findings of PWML on the DWI or SWI sequence and the appearance, as well as the evolution of the lesions between both scans and the occurrence of additional lesions. Possible differences in clinical characteristics between the appearances were tested with one-way ANOVA tests. Next, since the presence of lesions on DWI can be largely influenced by the time of scanning, day of life of the first MRI scan, defined as more or less than 10 days after birth, was included in two different logistic regression analyses along with cluster and/or linear appearance as independent variables and with the presence of abnormalities on DWI or SWI as dependent variables. Finally, both appearance and number of lesions were tested against outcome measures. For this linear regression analysis, the presence of additional lesions was also taken into account. A significance level of p<0.05 was used.

## Results

A total of 112 infants were eligible for the study. In 62 infants with a GA<28 weeks, MRIs were part of routine clinical care. For the other 50 infants, with a GA≥28 weeks, abnormal findings on cranial ultrasound examinations were the reason to perform an MRI. Both types of PWML were clearly seen on 1.5 Tesla and 3 Tesla magnets, although only a small group of infants was imaged on a 1.5 Tesla magnet. Clinical characteristics, general scan parameters and outcome data of all infants can be found in *Table 1*.

**Table 1:** Clinical characteristics.

Variable	Total	Linear	Cluster	Mixed	p-value
	*N = 112*	*N = 67*	*N = 26*	*N = 19*	
**Clinical parameters**					
Sex *(male/female)*	56/56 (50%)	34/33 (49%)	13/13 (50%)	9/10 (47%)	1.0
Gestational age *(weeks, mean [range])*	28.2 [24.4–33.4]	27.8 [24.4–33.4]	28.2 [24.7–31.6]	29.7 [26.1–32.7]	<0.01
Birth weight *(grams, mean [range])*	1158 [515–2100]	1101 [515–2100]	1164 [695–1940]	1354 [865–2100]	0.02
Birth weight z-score *(mean [range])*	0.4 [−1.7–2.0]	0.4 [−1.7–2.0]	0.4 [−1.6–1.5]	0.3 [−0.7–1.8]	0.9
Mechanical ventilation *(no/yes)*	33/79 (71%)	17/50 (75%)	9/17 (65%)	7/12 (63%)	0.5
Bronchopulmonary dysplasia *(no/yes)*	92/20 (18%)	54/13 (19%)	22/4 (15%)	16/3 (16%)	0.9
Patent ductus arteriosus *(no/yes)*	76/36 (32%)	44/23 (34%)	15/11 (42%)	17/2 (11%)	0.07
Necrotizing enterocolitis requiring surgical intervention *(no/yes)*	103/9 (8%)	63/4 (3%)	23/3 (12%)	17/2 (11%)	0.6
Culture proven sepsis *(no/yes)*	85/27 (24%)	51/16 (24%)	18/8 (31%)	16/3 (16%)	0.5
Intraventricular hemorrhage (no/grade I/grade II/grade III/grade IV)	56/7/15/12/22 (50%)	31/4/8/9/15 (54%)	18/1/3/2/2 (31%)	7/2/4/1/5 (63%)	0.08
Posthemorrhagic ventricular dilatation requiring intervention *(no/yes)*	95/17 (15%)	53/14 (21%)	26/0 (0%)	16/3 (16%)	0.04
**Scan parameters**					
Postmenstrual age at first scan *(weeks, mean [range])*	31.2 [26.6–34.7]	31.0 [26.6–34.7]	31.2 [29.6–33.7]	31.8 [29.6–34.6]	0.1
Postmenstrual age at TEA scan *(weeks, mean [range])*	41.2 [40.0–43.6]	41.2 [40.0–42.9]	41.1 [40.1–43.1]	41.1 [40.0–43.6]	0.7
Postnatal age at first scan *(days)*	21 [0–50]	22 [0–50]	21 [5–44]	14 [2–44]	0.04
Lesions besides PWML *(no/yes)*	67/45 (40%)	35/32 (48%)	22/4 (15%)	10/9 (47%)	0.01
PWML on first scan *(no/yes)*	21/91 (81%)	16/51 (76%)	3/23 (79%)	2/17 (90%)	0.2
PWML on TEA scan *(no/yes)*	36/76 (68%)	28/39 (58%)	8/18 (69%)	0/19 (100%)	<0.01
PWML on both scans *(no/yes)*	57/55 (49%)	44/23 (34%)	11/15 (58%)	2/17 (90%)	<0.01
**Follow-up parameters**					
Corrected age at 1^st^ visit *(months, mean [range])*	16.2 [12.6–21.0]	16.1 [12.6–20.4]	16.5 [13.8–19.4]	16.3 [14.7–21.0]	0.7
	N = 100	N = 59	N = 24	N = 17	
Griffiths developmental quotient 1^st^ visit *(mean [SD])*	102 [9]	102 [8] #	103 [8]	102 [11]	0.8
Corrected age at 2^nd^ visit *(months, mean [range])*	25.3 [22.6–31.3]	25.6 [23.7–31.3]	25.4 [23.2–30.1]	24.1 [22.6–26.4]	0.1
	N = 83	N = 52	N = 18	N = 13	
Griffiths developmental quotient 2^nd^ visit *(mean [SD])*	99 [14]	93 [9]	117 [17]	93 [5]	0.004
	N = 17	N = 7	N = 4	N = 6	
Bayley cognitive composite score 2^nd^ visit *(mean [SD])*	105 [13]	104 [13]	106 [15]	108 [10]	0.6
	N = 65	N = 44	N = 14	N = 7	
Bayley total motor composite score 2^nd^ visit *(mean [SD])*	108 [12]	108 [10]	110 [16]	106 [14]	0.7
	N = 65	N = 41∧	N = 13∧	N = 7	
Cerebral palsy (no/yes)	103/9	55/4	22/4	18/1	0.01
*With c-PVL*	1	0	1	0	
*With PVHI*	4	3	0	1	
*With PHVD*	1	1	0	0	
*Without additional lesions*	3	0	3	0	

Infants are scored according to the appearance of their lesions on the early scan. For the 21 infants that only showed lesions at TEA, the appearance at TEA is used.

Abbreviations: c-PVL  =  cystic periventricular leukomalacia; N =  number of patients; PVHI =  periventricular hemorrhagic infarction; PHVD =  post-hemorrhagic ventricular dilatation; PWML =  punctate white matter lesions; SD =  standard deviation; TEA =  term equivalent age.

# In one patient, with a hemiplegia, the Griffiths test was not completed.

∧In four patients (three with linear lesions, one with cluster lesions) the motor subtest of the Bayley scales could not reliably be completed.

### Appearance

An overview of appearances, location, laterality and lesion load is given in *Table 2* and of the findings on DWI and SWI in *Table 3* *,* and in more detail in *Table S1*.

**Table 2:** PWML characteristics.

	Appearance			Location			Laterality		Lesion load		
	*Linear*	*Cluster*	*Mixed*	*Anterior-mid*	*Posterior*	*Overall*	*Unilateral*	*Bilateral*	*1*–*3*	*4*–*6*	*>6 or >5%*
**Early (n = 91)**	51 (56)	23 (25)	17 (19)	85 (93)	1 (1)	5 (6)	15 (16)	76 (84)	33 (36)	29 (32)	29 (32)
**TEA (n = 76)**	53 (70)	9 (12)	14 (18)	71 (93)	1 (1)	4 (5)	11 (14)	65 (86)	50 (66)		26 (34)

The values between brackets represent the percentages.

Abbreviations: n =  number of patients; PWML =  punctate white matter lesions; TEA =  term equivalent age. Overall  =  diffusely located lesions, i.e. in all 3 of the mentioned regions.

**Table 3:** PWML identification using additional imaging methods.

	Early MRI (n = 91)				Term equivalent MRI (n = 76)		
Appearance	*DWI+*	*SWI+*	*No SWI available*	*SWI and DWI+* *	*DWI+*	*SWI+*	*No SWI available*
***Linear***	6 (7)	29 (32)	22 (24)	3 (3)	0 (0)	22 (29)	27 (36)
***Cluster***	10 (11)	6 (7)	10 (11)	1 (1)	0 (0)	1 (1)	2 (3)
***Mixed***	11 (12)	11 (12)	4 (4)	6 (7)	1 (1)	6 (8)	3 (4)

The values between brackets represent the percentages.

*The patients included in this column are also included in the columns ‘DWI+’ and ‘SWI+’.

Abbreviations: DWI =  diffusion weighted imaging; n =  number of patients; PWML =  punctate white matter lesions; SWI =  susceptibility weighted imaging.

#### Linear PWML

The most common type of PWML was linear, in 56% of the early and 70% of the TEA scans (*Figure 1*). Lesions were often more clear on T2-weighted than T1-weighted imaging. Location of the lesions was anterior or adjacent to the ventricles in all infants. For lesions with low SI on SWI, there was a significant association with a linear appearance (p = 0.002) and with a scan beyond the first 10 days after birth (p = 0.02). At TEA, low SI lesions on SWI were less likely to have a cluster appearance (p = 0.004). The presence of an IVH was borderline significantly associated with a linear appearance (p = 0.047). Low SI lesions on early SWI were found more often in infants with a GA<28 weeks (p = 0.0007) but no relation between GA and linear lesions on either scan was found.

#### Cluster PWML

Clusters of solitary lesions were seen in 25% and 12% on the early and TEA MRI, respectively (*Figure 2*). In infants with a cluster appearance on the early MRI, MRI at TEA showed lesions that were generally more florid on T1-weighted imaging as compared to T2-weighted imaging. Location of the lesions was anterior or adjacent to the ventricles. For the early MRI, logistic regression analysis showed a significant correlation between lesions with restricted diffusion on DWI and both a cluster appearance (p = 0.001) and a scan within the first 10 days after birth (p<0.0001). The presence of an IVH was not associated with lesions in a cluster appearance (p = 0.3). On the early scan, infants with a GA≥28 weeks were more likely to have lesions in a cluster appearance (p = 0.006) and also more likely to have lesions with restricted diffusion on DWI (p<0.0001).

#### Combination of linear and cluster PWML

A combination of both types of lesions was seen in 19% and 18% on the early and TEA MRI, respectively. Location of the lesions was anterior or adjacent to the ventricles in most, but in 5 infants, all with a high lesion load, lesions were also found in the posterior part of the brain. No association with IVH was found in this group (p = 0.3). Infants with a GA≥28 weeks were more likely to have lesions in a mixed appearance on the early scan (p = 0.005).

### Evolution

PWML were seen in 91 infants on the early MRI. Of those 91, PWML were still visible in 55 infants at TEA. In more than half of the infants with the lowest lesion loads on the early scan, lesions could no longer be identified at TEA. The majority of these lesions had a linear appearance. In an additional 21 infants, PWML were only seen at TEA, with <6 lesions in 18 and ≥6 lesions in three. For all infants with a high lesion load on the early scan, PWML were still present at TEA. Lesion load was still high in 21 patients (75%). In the infants with the highest lesion loads, appearance of PWML was cluster or mixed in 79%. Lesions on both scans were more often identified in the group of infants with a GA≥28 weeks (p = 0.001, see also *Table 2*) and in infants with lesions with a mixed appearance (p<0.001).

**Figure 3:**
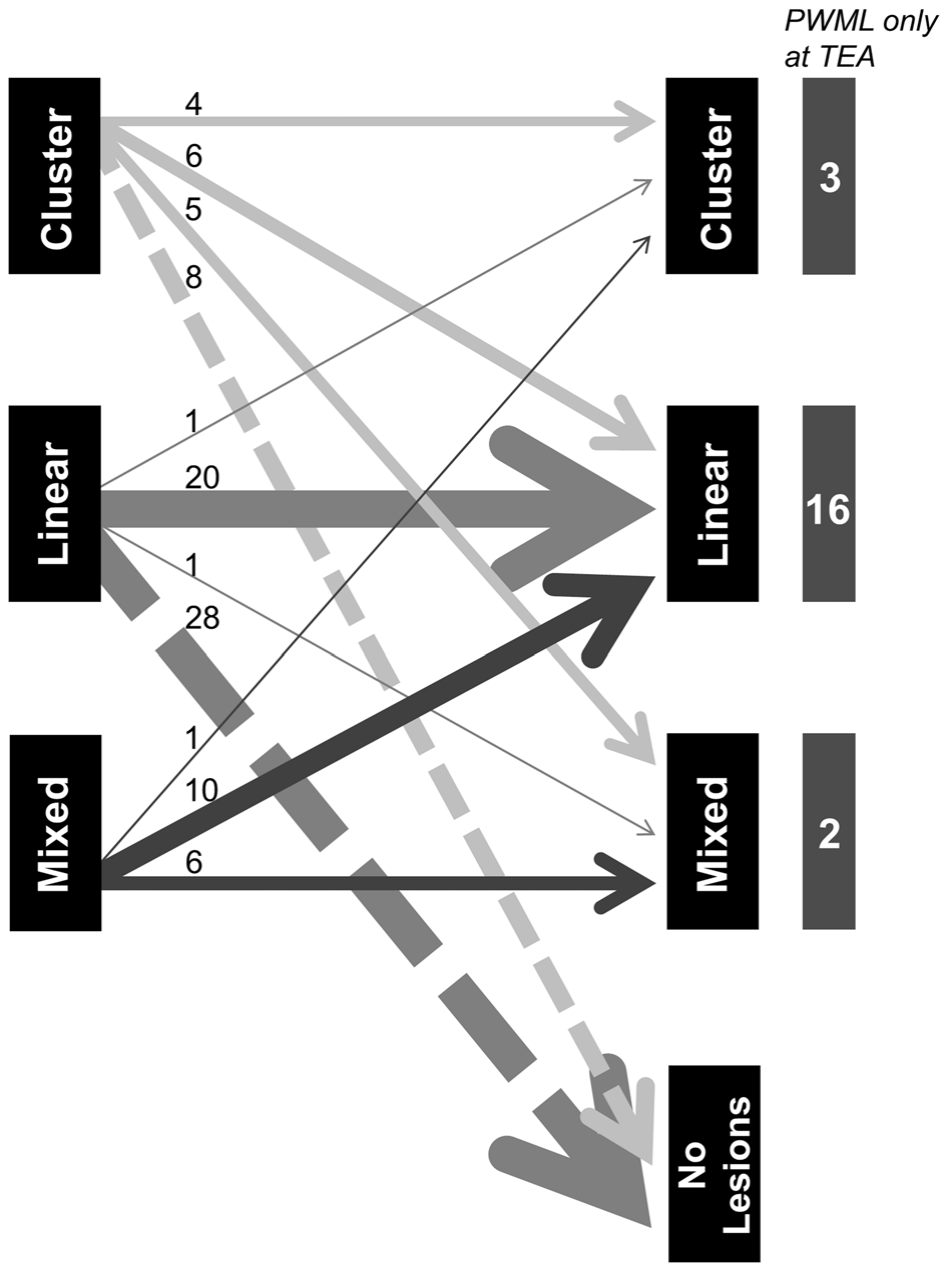
Changes in PWML appearance over time. This figure depicts the changes in appearance of PWML between both scans. Early imaging is represented on the left and term equivalent imaging on the right. The number of infants per category is depicted by the thickness of the arrow and also printed above each arrow. Especially cluster and mixed lesions will change appearance, whereas linear lesions remain linear in the majority of the infants, or are no longer visible at TEA. At the right side of the figure, infants are depicted that only showed PWML at TEA.

In the group of 55 infants with PWML on both scans, appearance differed between the scans in 24, as represented in *Figure 3*. Linear lesions only changed appearance in two out of 22 infants. One infant had only one lesion left at TEA and was thus scored as solitary and the other suffered from a parechovirus infection two weeks before his TEA scan, and showed lesions with a mixed appearance [16]. This was the only infant with lesions with restricted diffusion on DWI at TEA. In the majority of infants (85%) PWML were bilateral at both time points. Infants with unilateral lesions had either a very low lesion load or a unilateral PVHI with PWML on the contralateral side.

### Additional lesions

Additional lesions were identified in 45 infants. These were post-hemorrhagic ventricular dilatation in 8, PVHI in 25, cystic periventricular leukomalacia in 1, significant cerebellar hemorrhage in 7, lenticulostriate infarction in 3, and an arachnoid cyst in 1 infant. More lesions were found in the group with a GA≥28 weeks (p = 0.002), mainly due to the higher incidence of PVHI. This can be explained by the difference in inclusion criteria. Infants with lesions in a cluster appearance less often showed additional lesions compared to the other two groups (p = 0.01).

### Neurodevelopmental outcome

Follow-up data at 15 months corrected age were available for 100 infants. Seven infants were not seen and the remaining five were not yet 15 months of age. Follow-up was available for 83 infants around two years corrected age. Six infants were lost to follow-up and the remaining 23 were not yet two years of age. Outcome data are presented in *Table 1*. The majority of infants performed within the normal range (defined as above -1 standard deviation) on both motor and cognitive outcome. Both the relation between initial appearance (i.e., linear or cluster) and between lesion load (i.e., <or ≥6 lesions) and neurodevelopmental outcome as tested with the Bayley or Griffiths scales were explored. The only significant relation found was between a cluster appearance and a higher developmental quotient on the Griffiths scales at 24 months corrected age (p = 0.004). However, numbers were really small and this relation was skewed due to one outlier, an infant with a developmental quotient of 135. After exclusion of this infant, no significant differences were found.

**Figure 4:**
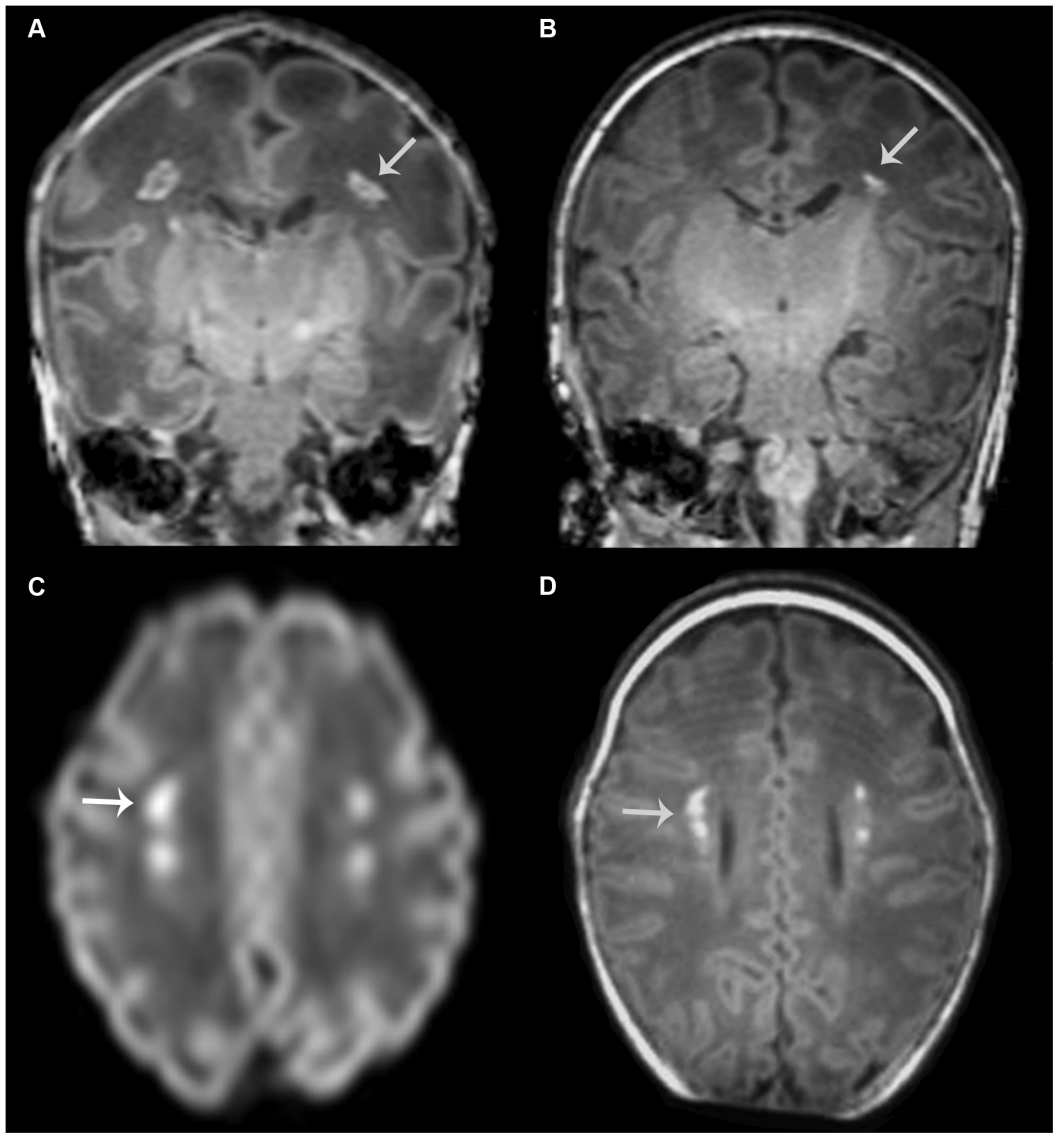
PWML in an infant without additional lesions who developed cerebral palsy. T1-weighted images at early (born at 31 weeks, postmenstrual age at scan 33 weeks, (A)) and term equivalent age (B) in an infant with lesions in a cluster pattern. Note that the lesions seem to be directly in the path of the corticospinal tracts (see arrow for an example). Lesions show high signal intensity, suggestive of restricted diffusion, on DWI of the early scan (C) and these are visible in the same location on T1-weighted imaging at term equivalent age (D). No signal intensity changes were seen on SWI on either scan and no additional lesions were identified. At term equivalent age, the posterior limb of the internal capsule appears to be less well developed on the right side. This infant subsequently developed a mild asymmetric bilateral spastic cerebral palsy, with her left leg being most severely affected.

Nine infants developed mild CP. Seven had an asymmetrical posterior limb of the internal capsule on T1-weighted imaging at TEA and six had additional overt white matter injury. All three infants without additional lesions who did develop CP had a cluster appearance on their early MRI, with a high lesion load (*Figure 4*). Lesions showed restricted diffusion on DWI in all. In two infants SWI was available, both without abnormalities. At TEA, lesions converted to a mixed appearance in two and a linear appearance in one, and lesion load was still high in all.

## Discussion

This is, to the best of our knowledge, the largest study so far describing sequential data of PWML in preterm infants and the first to make a distinction between two patterns of PWML with differences in appearance and evolution over time. These differences correlate with findings on DWI and SWI, with a relation between a cluster appearance and restricted diffusion on DWI, and a linear appearance and lesions with low SI on SWI.

The first *linear* pattern shows an often mild lesion load and lesions with low SI on SWI. The second *cluster or mixed* pattern shows more often a high lesion load, lesions without low SI on SWI and, depending on the time of scanning, restricted diffusion on DWI. In the infants with a GA≥28 weeks, the cluster and mixed patterns were more often identified compared to the linear pattern.

These two patterns may reflect differences in underlying pathology. In this study, lesions with a cluster appearance, similar to most lesions described in the literature, were found to have normal SI on SWI, suggesting a non-hemorrhagic origin. When imaged within the first 2 weeks after birth, DWI findings often showed high SI with concurrent low SI on the apparent diffusion coefficient map, suggesting an underlying restricted diffusion, with a possible inflammatory or ischemic origin. At TEA, lesions with a cluster appearance on the early MRI were most florid on T1-weighted imaging and generally more florid compared to lesions with a linear appearance. This is in agreement with lesions described in some of the previous studies with imaging at TEA, where lesions were also described to be most florid on T1-weighted imaging, possibly due to early gliosis [3], [7]. In contrast, lesions with a linear appearance were often found to have low SI on SWI, suggestive of a hemorrhagic origin. Only linear punctate lesions adjacent to the medullary veins in the white matter, suggesting more than just venous congestion, were scored. These linear lesions were seen more clearly on T2, especially on early imaging. This may be due to hemoglobin degradation products, which cause T2 shortening before they cause T1 shortening [5], [17]. Again, this is in agreement with some of the previous studies [5], [12], [18]. A few studies have already described a difference in types of PWML and our data confirm these findings [11], [19].

Although findings on DWI are limited to the early scan, since a restricted diffusion will only be visible during the first 10 to 14 days after injury, findings on SWI were visible on both scans. Often, lesions were better visible on SWI and sometimes more lesions were seen, not recognized on T1 or T2. Since restricted diffusion on DWI can also be caused by signal distortion due to hemorrhage, the combination of both DWI and SWI will be most informative. Therefore, these sequences should become part of the standard protocol when imaging very preterm infants.

In agreement with previous studies, lesion load differed between the two scans [1], [6], [20]. Although the majority of the lesions, especially those with a high lesion load, were already visible on the early MRI, lesions had resolved in 33% of the infants and were first seen on the TEA scan in 20%. Lesion load was generally lower in the infants with a GA<28 weeks, whose early scan was performed later after birth compared to the older age group. Some lesions could already have resolved before the early scan. Six out of 21 infants in whom lesions appeared between the two scans had a sepsis or underwent surgery in the period between both scans, which may be a possible explanation, comparable to late-onset cystic periventricular leukomalacia [21]. Of these six infants, four showed a linear, one a cluster and one a mixed pattern of PWML. In infants with a high lesion load, lesions were often less clear or decreased in number between the scans, with a milder pattern at TEA compared to the first MRI. Also, as shown in *Figure 3*, the appearance of the lesions changed, especially for the lesions with a cluster and mixed appearance on the early scan. This evolution may be due to the tissue reaction or partial resolution of the lesions. These two findings suggest that serial MR imaging is important to be able to identify the full extent and initial pattern of PWML, with the first MRI showing the full extent of the PWML and the second scan the evolution and distribution in relation to the corticospinal tracts.

Most lesions in this study were located adjacent to the ventricles, in the corona radiata and centrum semiovale. Only five infants, with a high lesion load, also showed lesions in the posterior white matter or along the optic radiation. This is somewhat different from previous studies, where posterior lesions were reported in 23–53% of cases [5], [20], [22]. A clear explanation for this difference could not be found.

Due to the heterogeneity in inclusion criteria, neurodevelopmental outcome data in infants with PWML differ between studies. High rates of CP have been reported in some studies [4], whereas others did not find this relation and described the presence and severity of additional lesions to be most indicative of an abnormal outcome at two years [5], [20]. Whether the presence of additional lesions was taken into account was not reported in all previous PWML related studies and this may explain at least some of the differences in outcome. In our study, six out of nine infants who developed CP had overt additional white matter lesions. In the other three, PWML are likely to be responsible for the development of their CP. Lesions in those infants appeared to be directly in the path of the corticospinal tracts and lesion load was high in all. Also, all lesions had a cluster appearance with restricted diffusion on DWI, suggesting ischemia as the underlying cause. None of the infants with a linear pattern developed CP.

PWML may have a more extensive effect on the development of the brain than can be seen on conventional imaging. Two studies have shown a lower fractional anisotropy in infants with PWML, suggesting an altered microstructure extending beyond the immediate area of injury, possibly indicating a more diffuse injury to these tracts which is not visible on conventional MRI [22], [23]. Sie and colleagues described a follow-up imaging study in infants with PWML on neonatal imaging, who were scanned again at 18 months of age. When only a few PWML were present at TEA, no abnormalities on imaging were seen at 18 months and those children all had a normal neurodevelopmental outcome. However, in the presence of more than 6 lesions at TEA, the MRI at 18 months showed gliosis at the site of the lesions and some of those infants developed CP, similar to the findings in this study [18]. Lesion load may therefore be important for prediction of neurodevelopmental outcome. Outcome data beyond two years of age are currently lacking. Several authors have speculated that PWML may be related to the milder forms of cognitive and behavioral problems found at school age in preterm infants and this seems a likely hypothesis [5], [18], [20].

There are several limitations to this study. First, due to the retrospective nature of our study, our cohort consisted of two subgroups. All infants with a GA<28 weeks were scanned, whereas those ≥28 weeks were only scanned when cranial ultrasound examination was indicative of white matter injury, most often inhomogeneous periventricular echogenicity or overt white matter pathology, such as PVHI. Therefore, we may well have missed the mild lesions in infants ≥28 weeks gestation which may have skewed our findings. Also, infants <28 weeks were scanned around 30 weeks postmenstrual age and thus beyond the optimal timing to see restricted diffusion on DWI.

Thirdly, SWI was introduced during the study period and was not done in all infants, limiting the number of infants in which a comparison between DWI and SWI could be made. Finally, outcome data are not yet available for the total cohort and are restricted to two years of age.

In conclusion, two different patterns of PWML were seen in a large population of serially scanned preterm infants on T1-weighted and T2-weighted imaging, with discordant findings on DWI and SWI. These two patterns are suggestive of a difference in underlying pathology. Evolution of the lesions between the two scans often showed a decrease in lesion load and a change in appearance of the lesions. An early scan is therefore needed to be informed about the full extent of the lesion load. For a more complete classification of PWML, both conventional imaging and the combination of DWI and SWI are required. Long-term follow-up studies are needed to determine whether this difference in patterns will also explain differences in outcome.

## Supporting Information

Table S1 **PWML imaging characteristics of the total cohort, and separately for infants above and below 28 weeks of gestation.**
(DOCX)

## References

[1] Debillon T, N'Guyen S, Muet A, Quere MP, Moussaly F, et al (2003) Limitations of ultrasonography for diagnosing white matter damage in preterm infants. Arch Dis Child Fetal Neonatal Ed 88: F275–F279. 12819157 10.1136/fn.88.4.F275 PMC1721566

[2] Maalouf EF, Duggan PJ, Counsell SJ, Rutherford MA, Cowan F, et al (2001) Comparison of findings on cranial ultrasound and magnetic resonance imaging in preterm infants. Pediatrics 107: 719–727. 11335750 10.1542/peds.107.4.719

[3] Miller SP, Cozzio CC, Goldstein RB, Ferriero DM, Partridge JC, et al (2003) Comparing the diagnosis of white matter injury in premature newborns with serial MR imaging and transfontanel ultrasonography findings. AJNR Am J Neuroradiol 24: 1661–1669. 13679289 PMC7973994

[4] de Bruine FT, van den Berg-Huysmans AA, Leijser LM, Rijken M, Steggerda SJ, et al (2011) Clinical implications of MR imaging findings in the white matter in very preterm infants: a 2-year follow-up study. Radiology 261: 899–906. 22031710 10.1148/radiol.11110797

[5] Cornette LG, Tanner SF, Ramenghi LA, Miall LS, Childs AM, et al (2002) Magnetic resonance imaging of the infant brain: anatomical characteristics and clinical significance of punctate lesions. Arch Dis Child Fetal Neonatal Ed 86: F171–F177. 11978747 10.1136/fn.86.3.F171 PMC1721406

[6] Dyet LE, Kennea N, Counsell SJ, Maalouf EF, Ajayi-Obe M, et al (2006) Natural history of brain lesions in extremely preterm infants studied with serial magnetic resonance imaging from birth and neurodevelopmental assessment. Pediatrics 118: 536–548. 16882805 10.1542/peds.2005-1866

[7] Leijser LM, de Bruine FT, Steggerda SJ, van der Grond J, Walther FJ, et al (2009) Brain imaging findings in very preterm infants throughout the neonatal period: part I. Incidences and evolution of lesions, comparison between ultrasound and MRI. Early Hum Dev 85: 101–109. 19144474 10.1016/j.earlhumdev.2008.11.010

[8] Leijser LM, Steggerda SJ, de Bruine FT, van der Grond J, Walther FJ, et al (2009) Brain imaging findings in very preterm infants throughout the neonatal period: part II. Relation with perinatal clinical data. Early Hum Dev 85: 111–115. 19135814 10.1016/j.earlhumdev.2008.11.012

[9] McCrea HJ, Ment LR (2008) The diagnosis, management, and postnatal prevention of intraventricular hemorrhage in the preterm neonate. Clin Perinatol 35: 777–92, vii. 10.1016/j.clp.2008.07.014 PMC2901530 19026340

[10] Benders MJNL, Kersbergen KJ, de Vries LS (2014) Neuroimaging of White Matter Injury, Intraventricular and Cerebellar Hemorrhage. Clin Perinatol 41: 69–82. 24524447 10.1016/j.clp.2013.09.005

[11] Rutherford MA, Supramaniam V, Ederies A, Chew A, Bassi L, et al (2010) Magnetic resonance imaging of white matter diseases of prematurity. Neuroradiology 52: 505–521. 20422407 10.1007/s00234-010-0700-y

[12] Niwa T, de Vries LS, Benders MJ, Takahara T, Nikkels PG, et al (2011) Punctate white matter lesions in infants: new insights using susceptibility-weighted imaging. Neuroradiology 53: 669–679. 21553013 10.1007/s00234-011-0872-0 PMC3156303

[13] de Bruine FT, Steggerda SJ, van den Berg-Huysmans AA, Leijser LM, Rijken M, et al (2014) Prognostic value of gradient echo T2* sequences for brain MR imaging in preterm infants. Pediatr Radiol 44: 305–312. 24419491 10.1007/s00247-013-2803-1

[14] Griffiths R. (1984) The abilities of young children. A comprehensive system of mental measurement for the first eight years of life. London: The test agency Ltd.

[15] Bayley N. (2006) Bayley Scales of Infant and Toddler Development, 3rd edition. San Antonio, TX: Harcourt Assessment. 10.1016/j.ridd.2013.07.006 24029804

[16] Verboon-Maciolek MA, Krediet TG, Gerards LJ, de Vries LS, Groenendaal F, et al (2008) Severe neonatal parechovirus infection and similarity with enterovirus infection. Pediatr Infect Dis J 27: 241–245. 18277927 10.1097/INF.0b013e31815c1b07

[17] Gomori JM, Grossman RI, Goldberg HI, Zimmerman RA, Bilaniuk LT (1985) Intracranial hematomas: imaging by high-field MR. Radiology 157: 87–93. 4034983 10.1148/radiology.157.1.4034983

[18] Sie LT, Hart AA, van HJ, de GL, Lems W, et al (2005) Predictive value of neonatal MRI with respect to late MRI findings and clinical outcome. A study in infants with periventricular densities on neonatal ultrasound. Neuropediatrics 36: 78–89. 15822020 10.1055/s-2005-837574

[19] Raybaud C, Ahmad T, Rastegar N, Shroff M, Al NM (2013) The premature brain: developmental and lesional anatomy. Neuroradiology 55 Suppl 223–40. 23832006 10.1007/s00234-013-1231-0

[20] Miller SP, Ferriero DM, Leonard C, Piecuch R, Glidden DV, et al (2005) Early brain injury in premature newborns detected with magnetic resonance imaging is associated with adverse early neurodevelopmental outcome. J Pediatr 147: 609–616. 16291350 10.1016/j.jpeds.2005.06.033

[21] Andre P, Thebaud B, Delavaucoupet J, Zupan V, Blanc N, et al (2001) Late-onset cystic periventricular leukomalacia in premature infants: a threat until term. Am J Perinatol 18: 79–86. 11383704 10.1055/s-2001-13633

[22] Bassi L, Chew A, Merchant N, Ball G, Ramenghi L, et al (2011) Diffusion tensor imaging in preterm infants with punctate white matter lesions. Pediatr Res 69: 561–566. 21386750 10.1203/PDR.0b013e3182182836

[23] Miller SP, Vigneron DB, Henry RG, Bohland MA, Ceppi-Cozzio C, et al (2002) Serial quantitative diffusion tensor MRI of the premature brain: development in newborns with and without injury. J Magn Reson Imaging 16: 621–632. 12451575 10.1002/jmri.10205

