# Correction: Different Patterns of Punctate White Matter Lesions in Serially Scanned Preterm Infants

**DOI:** 10.1371/journal.pone.0114704

**Published:** 2014-11-26

**Authors:** The PLOS ONE Staff

An affiliation for the 2^nd^ author is missing. Manon J. N. L. Benders is also affiliated with: Centre for the Developing Brain, Division of Imaging Sciences and Biomedical Engineering, King's College London and King's Health Partners, St. Thomas' Hospital, London, United Kingdom.
