# Supplementary material for: Different Patterns of Punctate White Matter Lesions in Serially Scanned Preterm Infants
Source: PLoS One. 2014 Oct 3;9(10):e108904. doi: 10.1371/journal.pone.0108904 (PMC4184838; doi:10.1371/journal.pone.0108904)
Supplement: Table S1 — PWML imaging characteristics of the total cohort, and separately for infants above and below 28 weeks of gestation. (DOCX) [file pone.0108904.s001.docx]

***Table S1. PWML imaging characteristics of the total cohort, and separately for infants above and below 28 weeks of gestation***

|  | **Appearance** | |  | |  | **Location** | |  |  | | **Laterality** | |  | **Lesion load** | |  | | |  |
| --- | --- | --- | --- | --- | --- | --- | --- | --- | --- | --- | --- | --- | --- | --- | --- | --- | --- | --- | --- |
|  | ***Linear*** | ***Cluster*** | | ***Mixed*** | | ***Anterior-mid*** | ***Posterior*** | | | ***Overall*** | ***Unilateral*** | ***Bilateral*** | | ***1-3*** | ***4-6*** | | | ***>6 or >5%*** | |
| **Early (n=91)** | 51 (56) | 23 (25) | | 17 (19) | | 85 (93) | 1 (1) | | | 5 (6) | 15 (16) | 76 (84) | | 33 (36) | 29 (32) | | | 29 (32) | |
| **<28 wks (n=49)** | 34 (70) | 11 (22) | | 4 (8) | | 49 (100) | 0 (0) | | | 0 (0) | 8 (16) | 41 (84) | | 30 (61) | 12 (24) | | | 7 (14) | |
| **≥28 wks (n=42)** | 17 (40) | 12 (29) | | 13 (31) | | 36 (86) | 1 (2) | | | 5 (12) | 7 (17) | 35 (83) | | 3 (7) | 17(40) | | | 22 (52) | |
| **TEA (n=76)** | 53 (70) | 9 (12) | | 14 (18) | | 71 (93) | 1 (1) | | | 4 (5) | 11 (14) | 65 (86) | | 50 (66) | | |  | 26 (34) | |
| **<28wks (n=35)** | 26 (74) | 5 (14) | | 4 (11) | | 34 (97) | 0 (0) | | | 1(3) | 7 (20) | 28 (72) | | 31 (89) | | |  | 4 (10) | |
| **≥28 wks (n=41)** | 27 (66) | 4 (10) | | 10 (24) | | 37 (90) | 1 (2) | | | 3 (7) | 4 (10) | 37 (90) | | 19 (46) | | |  | 22 (54) | |

*The values between brackets represent the percentages*
